# New Insights into the Role of the Complement System in Human Viral Diseases

**DOI:** 10.3390/biom12020226

**Published:** 2022-01-28

**Authors:** Ewa Ostrycharz, Beata Hukowska-Szematowicz

**Affiliations:** 1 Institute of Biology, University of Szczecin, 71-412 Szczecin, Poland; ewa.ostrycharz@phd.usz.edu.pl; 2 Doctoral School of the University of Szczecin, University of Szczecin, 71-412 Szczecin, Poland; 3 Molecular Biology and Biotechnology Center, University of Szczecin, 71-412 Szczecin, Poland

**Keywords:** complement system, viral diseases, respiratory diseases, COVID-19, SARS, MERS, acute liver failure, HB, disseminated intravascular coagulation, vector-borne diseases

## Abstract

The complement system (CS) is part of the human immune system, consisting of more than 30 proteins that play a vital role in the protection against various pathogens and diseases, including viral diseases. Activated via three pathways, the classical pathway (CP), the lectin pathway (LP), and the alternative pathway (AP), the complement system leads to the formation of a membrane attack complex (MAC) that disrupts the membrane of target cells, leading to cell lysis and death. Due to the increasing number of reports on its role in viral diseases, which may have implications for research on severe acute respiratory syndrome coronavirus 2 (SARS-CoV-2), this review aims to highlight significant progress in understanding and defining the role of the complement system in four groups of diseases of viral etiology: (1) respiratory diseases; (2) acute liver failure (ALF); (3) disseminated intravascular coagulation (DIC); and (4) vector-borne diseases (VBDs). Some of these diseases already present a serious global health problem, while others are a matter of concern and require the collaboration of relevant national services and scientists with the World Health Organization (WHO) to avoid their spread.

## 1. Introduction

### 1.1. The Complement System (CS)

**Figure 1:**
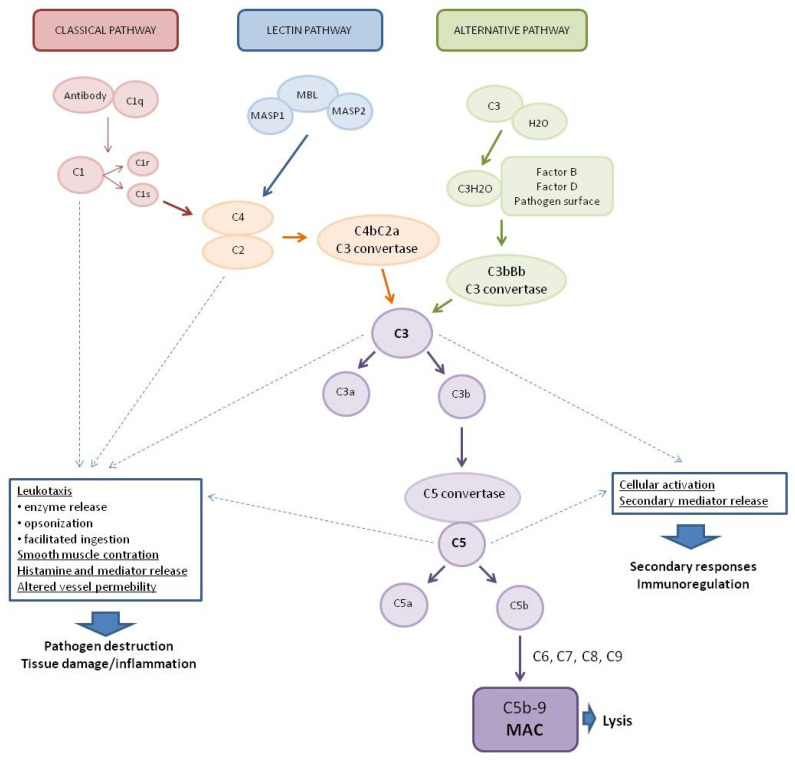
Pathways of complement system activation. MASP1—mannan-binding lectin associated serine protease-1, MASP2—mannan-binding lectin associated serine protease-2, MBL—mannose-binding lectin, and MAC—membrane attack complex.

The complement system (CS) plays a key role in the defense against pathogens as part of the human immune system [1,2]. It consists of more than 30 soluble proteins synthesized mainly by the liver (but also by leukocytes), which are present in the plasma in an inactive form [3], as well as membrane-bound regulators and receptors that interact with various cells and mediators of the immune system [4]. Activation of the complement system involves a cascade of enzymatic and non-enzymatic reactions that culminate in opsonization by various opsonins (e.g., C3b and C4b) of the pathogens or pathogen-virus-infected cells and then lysis of these cells by a set of proteins that form a membrane attack complex (MAC) [5,6]. In addition, activation of the complement system leads to the production of anaphylatoxins—potent proinflammatory molecules. Complement also served to mediate clearance of immune complexes and damaged self cells or cell debris and to mediate phagocytosis by neutrophils and monocytes [7,8]. A limited number of reports indicate that complement may contribute to the regulation of the anti-inflammatory response. This process involves T lymphocytes (Treg), crucial in the production of anti-inflammatory cytokines such as transforming growth factor-β (TGF-β), IL-10, and IL-35 [8]. The system is activated by three main pathways: the classical pathway (CP), the lectin pathway (LP), and the alternative pathway (AP) (Figure 1) [9].

The classical complement system pathway is referred to as “antibody-dependent” because of the involvement of IgM/IgG antibodies in the activation. Protein C1q (part of the C1 complex which consists of six molecules of C1q, two molecules of C1r, and two molecules of C1s) binds to the Fc region of complement-fixing antibodies (generally IgG1 and IgM) attached to pathogenic surfaces and pathogen-infected cells. This results in the activation of C1r and C1s proteases in the C1 complex [10,11]. C1s cleaves C4 and C2 proteases into large fragments (C4b, C2a) and small fragments (C4a, C2b). The larger fragments combine to form the C4bC2a complex on the pathogen surface which leads to the cleavage of C3 into anaphylatoxin C3a and opsonin C3b. The production of C3 convertase is the point where three complement pathways converge and afterwards have common steps to form MAC [1]. The lectin complement system pathway is similar to the classical pathway, but is independent of immunoglobulins. It does not recognize antigen antibody complexes but employs germline-encoded pattern-recognition receptors (PRRs) such as mannose-binding lectin (MBL) and ficolins [5]. When MBL recognizes and binds to carbohydrates in pathogen-associated molecular patterns (PAMPs), such as those found in viruses [12], MBL-associated serine proteases (MASPs, MBL-associated serine protease-2 (MASP-2), and MBL-associated serine protease-1 (MASP-1)) are activated to cleave complement components C2 and C4, which then leads to the generation of C3 convertase [13,14,15]. The alternative complement system pathway, in contrast to the classical and lectin pathways, consists of three processes that partially overlap. In the alternative pathway, activators include viruses [16]. Activation of the C3 convertase in this pathway occurs slowly in plasma and lead to the formation of C3H2O. C3 activation is induced by the presence of various surfaces which lack complement regulatory proteins which adsorb C3 to the surface to induce its conformational changes. C3H2O can then bind factor B (FB) to induce another conformational change as FB is cleaved into two components (FBb and FBa) by factor D (FD) [5]. This convertase begins to cleave C3 into C3a and C3b, in a manner analogous to the C4bC2a convertase in the classical and lectin pathway. The resulting C3b can bind to cell surfaces and FB to form the predominant convertase in the alternative pathway, i.e., C3bBb [2,17]. This C3bBb complex can be further stabilized by properdin, protecting it from the inactivating factor H (FH) and factor I (FI) [18]. All surface-bound C3 convertases, regardless of their origin, can induce an amplification branch of the alternative complement system pathway through the activation of C3 [19], which increases the density of deposited C3b and gradually leads to the formation of convertases that contain an additional C3b molecule (C4b2b3b or C3bBb3b) shifting its substrate specificity towards C5. The C5 convertase of the alternative pathway cleaves C5 into the anaphylatoxin C5a and the C5b fragment. When C5b binds to C6 and C7, the complex is inserted into cell membranes and interacts with C8, inducing the binding of several C9 units to form the MAC complex C5b6789 [20].

Activation of the complement system, regardless of the pathway, results in the generation of three broad effector pathways that enable the complement system to perform its physiological functions in host defense: direct lysis of target surfaces by the MAC, alerting and stimulating the immune system by producing potent proinflammatory anaphylatoxins, and opsonization of target surfaces by opsonins C4b, C3b, and C3bi [2,5,17]. MAC formation and targeted lysis are important effectors of the complement system’s anti-pathogenic actions [9]. Some cleavage products and the complement system activation products can act as anaphylatoxins and have broader immune regulatory functions. Most notably, the cleavage products C3a and C5a can be generated by all three pathways and can act as potent immune regulators, whereas C4a is generated exclusively by the classical and lectin pathways [21]. C3a and C5a can affect chemotaxis of eosinophilia, fibroblasts, macrophages, mast cells, and monocytes to the site of infection and inflammation, C5a alone is responsible for neutrophil recruitment [6], while C4a acts as an effector protein and increases endothelial cell permeability and enhances stress fiber formation [22]. In addition to their roles in chemotaxis, C3a and C5a have been implicated in the regulation of vasodilation, increased vascular permeability [23], and the production of various cytokines, including IL-1β, IL-8/CXCL-8, CCL5, IL-6, and tumor necrosis factor-α (TNF-α) [24,25].

The complement system, with its ability to form channels in the cell membrane, induce phagocytosis, and cause mast cell degranulation, is a dangerous weapon, and without constant supervision by the various mechanisms regulating its activity (Table 1), it could easily lead to cell and tissue damage in our body.

### 1.2. The Complement System at the Crossroads of the Innate and Adaptive Immune Response

The complement system is a part of the immune system and plays the role of a functional bridge between innate and adaptive immune responses that allows an integrated host defense against pathogens [9]. The humoral immune response is designed to protect extracellular spaces by activating effector and memory B cells and producing antibodies, leading to the neutralization and opsonization of the pathogen and providing immune memory against reinfection [5]. Complement system effectors are involved in humoral responses at multiple stages of B lymphocyte differentiation [35]. The complement system enhances B cell immunity mainly through complement receptors (CR), complement receptor type 1 (CR1) (CD35), and complement receptor type 1 (CR2) (CD21) expressed on B lymphocytes and follicular dendritic cells (FDCs). The CR2 receptor forms a receptor complex with the signaling protein CD19 and protein CD81 to form a receptor complex (CD21-CD19-CD81) of B lymphocytes. This supports enhanced B cell receptor (BCR e.g., surface immunoglobulins)-mediated signaling upon encountering a pathogen coated with complement system opsonins, resulting in a lowered threshold for B lymphocyte activation [36,37]. Coupling C3d to a low-affinity antigen which in the absence of coupling would cause B cell death, results not only in survival but also in B cell activation and antibody production [38]. Furthermore, the CR2 receptor mediates antigen-independent signals that are essential for B cell survival [39].

A regulatory effect on B lymphocytes is also induced by anaphylatoxins. Anaphylatoxin C3a causes the suppression of polyclonal B lymphocyte responses, while C5a promotes naive B cell migration and memory [40,41]. The complement system also influences T cell-associated responses. Experiments on mice lacking complement system inhibitory proteins (Decay-accelerating Factor (DAF) and CD59) highlight the important regulatory role of the complement system in the development of T cell immunity [9,42]. DAF deficiency increases cytokine production by T cells, and CD59 ligation decreases CD4+ T cell activation [43]. The interaction between antigen-presenting cells (APC) and T lymphocytes induces the local production of C3, C5, as well as FB and FD. Moreover, C3aR and C5aR receptors are upregulated on T lymphocytes, whereas DAF production is downregulated. Local production of complement system components from immune cells allows signals to be transduced by C3aR and C5aR receptors in an autocrine and paracrine manner. Complement system component C3 activated in the alternative pathway can increase the production of proinflammatory cytokines from T cells [44,45]. Induction of Th1 responses also depends on the activation of C3aR and CD46 receptor on T cells through their T cell-derived ligands [46]. In contrast, the absence of C3aR and C5aR receptors leads to reduced complement protein and receptor regulation, lack of expression of co-stimulatory molecules, impaired production of cytokines (IL-1, IL-23, and IL-12), induction of the Treg cell response, and inhibition of T cell proliferation [47,48,49].

### 1.3. Antiviral Activity of the Complement System and Viral Strategies for Reducing the Complement System Action

All three complement system pathways can lead to viral opsonization and deposition of complement system components upon activation. The outcome of this response is highly dependent on the infectious agent and can enhance viral infection, suppress viral infection, or be dysregulated by the expression of certain viral proteins [50]. The MBL protein of the lectin pathway can interact with numerous viral antigens and have different effects on neutralization or increased viral replication. MBL can directly bind the glycoprotein (GP) of the Ebola virus (EBOV) [51]. High doses of MBL, relative to other complement proteins, can enhance infection with nonreplicating (pseudotyped) EBOV-GP virus in primary human macrophages and human monocyte-derived macrophage cell lines [51]. This may be due to the activation by viral ligands of the C1QBP (gC1qR) translocation, which inhibits RIG-1 and then inhibits antiviral signaling by downregulating type I interferons. It is speculated that EBOV-MBL complexes activate C1QBP, which then negatively regulates RIG-1 inhibition of viral infection, thereby enhancing viral proliferation [51]. Additionally, MBL opsonization of the EBOV GP prevents GP binding to DC-SIGN (dendritic cell-specific intercellular adhesion molecule) and, therefore, neutralizes EBOV (pseudotype) [52]. Thus, in the context of EBOV infection, MBL effects appear to be dependent on the cellular target and the relative concentrations of other complement system protein components. It has also been shown that in vitro infection by the human immunodeficiency virus (HIV) of CD4+ H9 lymphoblasts is inhibited by MBL from human serum. In addition, MBL is able to selectively bind to HIV-infected H9 cells and HIV-infected U937 cell line [53]. These results indicate that MBL inhibits viral entry to susceptible cells. Similarly, in another study [53], gp120 HIV bound directly to MBL. In a later study about HIV, MBL was shown to be sufficient for virus opsonization but not neutralization [54]. It also showed that both the primary isolates (PI) of HIV and cell line-adapted HIV, despite binding to MBL, are relatively resistant to neutralization by MBL at the levels normally present in the serum. However, binding and opsonization of HIV by MBL may alter virus trafficking and viral-antigen presentation during HIV infection [54]. In another study, ref. [55] complement opsonization of herpes simplex virus-2 (HSV-2) both by human serum and by seminal plasma, produced enhanced infection of DCs and resulted in greater productive infection compared to free, nonopsonized HSV-2. Furthermore, opsonization gave rise to significantly higher gene expression of all inflammatory (TNF-α, IL-6, IL-1β) and antiviral factors (IFN-α, IFN-β, MX1), but at the protein level these differences between free and complement-opsonized HSV-2 were not as clear as at the gene level. The enhanced infection induced by complement-opsonized virions required the functional complement receptor 3 (CR3). In contrast, the presence of complement in combination with HSV-1 or HSV-2-specific antibodies decreased infection, inflammation, and antiviral responses of DCs. HSV-2 infection of DCs required endocytosis and endosomal acidification, as inhibition of these cellular events decreased infection [55].

Other complement proteins and subsequently the complement system activation products can opsonize virus particles. In the case of dengue virus (DENV) and West Nile virus (WNV), viral neutralization occurs in a C3- and C4-dependent manner after MBL binding. In the case of WNV, neutralization was achieved despite reduced levels of C5, indicating that neutralization did not require MAC generation [56]. In the case of monkey simian virus 5 (SV5), complement-mediated neutralization is achieved mainly through C3 deposition and the formation of viral aggregates rather than viral lysis [57]. Similarly, the complement activation in the presence of Influenza A Virus (IVA) results in viral aggregation and opsonization of the hemagglutinin receptor, although IgM antibodies and activation of the classical pathway are required to achieve neutralization [58]. Some complement proteins may also have a protective intracellular function. Intracellular C3 signaling induces the production of proinflammatory cytokines (IFN-β, IL-6, and IL-1β) by nuclear factor kappa B (NF-κB), and activates interferon regulatory factor (IRF), and activator protein-1 (AP-1). Detection of intracellular C3 has been shown to be dependent on mitochondrial antiviral signaling protein (MAVS) and independent of PAMPs and pattern recognition receptors (PRRs) [59]. Infected host cells that present viral antigens on the cell surface membrane can activate the classical pathway as the antigens bind IgM/IgG, and induce complement-dependent cytotoxicity (CDC). The infected cell is then lysed by MAC to reduce the virus titer. However, some viruses have evolved self-defense mechanisms against the action of the complement system to enable their survival [50] (Table 2).

This review focuses on presenting progress in understanding and delineating the role of the complement system in several groups of diseases. These diseases annually threaten human life, and it is safe to say that they still represent an unmet medical need. The etiological agents of these diseases, the viruses, are the cause of the epidemics and pandemics and constitute a serious global health problem. The complement system, in the case of viral diseases, can act at different levels, including the destruction of pathogens and infected cells, and can also exacerbate the disease, contributing to overall morbidity. In the latter case, it is important to find new strategies to overcome the negative effects of the complement system in the course of a viral disease and dramatically improve clinical outcomes.

## 2. The Role of the Complement System in Viral Diseases

### 2.1. The Complement System in Respiratory Diseases

#### 2.1.1. The Complement System in Acute Lung Injury (ALI) and Acute Respiratory Distress Syndrome (ARDS)

Acute lung injury (ALI) and acute respiratory distress syndrome (ARDS) describe clinical acute respiratory failure (ARF) syndromes with high morbidity and mortality. They are acute, life-threatening, and inflammatory lung injuries manifested by hypoxia and lung stiffness due to increased pulmonary vascular permeability, which almost always requires mechanical ventilation support [72,73]. Sepsis, pneumonia, trauma, and multiple blood transfusions are responsible for the majority of cases developing ARDS [74]; however, viruses are now increasingly being highlighted as the etiopathogenesis of ALI/ARDS. The pathogenesis of ALI/ARDS includes an acute phase characterized by initial damage to the vascular endothelium and/or alveolar epithelium resulting in increased alveolar and capillary permeability and pulmonary edema, neutrophil accumulation, the release of proinflammatory cytokines including TNF-α, IL-1β, and IL-6, and the release of toxic proteases and reactive oxygen species [75]. Activation of the complement system is a potential common denominator in the pathogenesis of ARDS and ALI, including those of viral etiology.

Lipopolysaccharide (LPS)-induced lung injury in mice is one of the most robust experimental models used to study ALI and ARDS in humans. Clinical and experimental studies confirm the important role of the complement system activation [76]. Wang et al. [76] show the involvement of anaphylatoxin C5a and its C5a-like receptor 2 (C5L2) in ALI. In a mouse model of ALI associated with LPS administration, there was abundant alveolar hemorrhage and increased vascular permeability and neutrophil infiltration [77,78,79]. Activation of the complement system pathway by LPS and generation of C5a may contribute to lung injury [77]. Silencing of C5L2 (C5L2-/-) affected enhanced β-arrestin signaling mediated by C5aR [80] and increased numbers of macrophages and neutrophils in bronchoalveolar lavage fluid (BALF) [76]. Additionally, C5L2-/- mice exhibited significantly higher levels of neutrophil activation and degranulation compared to wildtype mice. Silencing of C5a-C5L2 signaling also led to increased pro-inflammatory cytokines (IL-6 and TNF-α) in BALF. Similar to inflammatory cell influx, C5L2-/- is associated with increased expression of inflammatory cytokine genes and chemokines (macrofage inflammatory protein-MIP-2α and MIP-3α). However, C5L2 deficiency is also associated with increased expression of suppressor of cytokine signaling proteins (SOCS) responsible for inhibiting the Janus kinase (JAK)-signal transducer and activator of transcription (STAT) pathway (JAK-STAT signaling pathway) and the immune-responsive gene 1 (IRG-1) [76]. Both gene (SOCS and IRG-1) products are associated with suppression or recovery of Toll-like receptor (TLR) responses [81,82].

Although it seems somewhat counterintuitive, C5L2 deficiency appears to enhance both the pro- and anti-inflammatory sides of LPS-mediated injury. Nevertheless, the histopathological picture of the lung in C5L2 deficiency is characterized by much greater damage and reduced lung function. Studies suggest that C5L2 may act as a negative modulator of C5a-C5aR signal transduction [76] and a study by Huber-Lang et al. [83] shows that C5L2 overexpression is positively correlated with human ARDS survival. In contrast, anti-C5aR administration in mice affects reduced neutrophilic inflammation in BALF, which also suggests the involvement of C5a-C5aR in lung failure [76]. Studies of the effect of the complement component C5 in ALI show that the C5 gene knockout (C5-/-) with mice induces reduced caspase-3 activity and thus reduced apoptosis in lung tissue [84]. No effect of C5 silencing has been observed on the increase in inflammatory factors (IL-6, monocyte chemoattractant protein-1 (MCP-1) also, known as chemokine CC-motif ligand 2 (CCL2), and granulocyte colony-stimulating factor (G-CSF)) in lung tissue compared to wildtype mice. However, C5 silencing resulted in increased levels of myeloperoxidase (MPO) activity suggesting only increased granulocyte degranulation in lung tissue and not increased numbers of granulocytes [84]. The lung damage in the course of ALI induced by LPS and IgG immune complexes was preceded by the production of C5a anaphylatoxin in the BALF and lung tissue. That study demonstrates the involvement of endogenous C5a in the development of ALI, as mice with a genetic deficiency of C5 were partially protected from LPS-induced ALI development [85]. In contrast, intratracheal administration of the recombinant mouse complement component C5a (rmC5a) causes alveolitis with abundant leukocyte recruitment to alveolar spaces and severe alveolar-capillary barrier dysfunction and white blood cell influx into the BALF. In contrast, administration of equivalent amounts of recombinant mouse complement component C3a or arginine-deficient C5a did not reproduce the ALI phenotype induced by rmC5a. It has also been reported that activated alveolar macrophages and granulocytes release serine proteases that cause local cleavage of C5 to C5a, thereby enhancing lung injury [86,87]. Studies indicate that simultaneous activation of the PI3K/Akt and MEK1/2 pathways, which modulate the cytokine response during inflammation, is required for the development of C5a-induced lung inflammation. BALF obtained from mice with ALI induced by rmC5a administration was characterized by elevated levels of IL-1β, IL-6, IL-12 (p40), chemokines (CCL3, CCL4, CCL5, CCL11), TNFα, G-CSF, and granulocyte-macrophate colony-stimulating factor (GM-CSF). In addition, C5a caused the release of the chemokines CCL3, CCL4, and CCL5, which bind to the same receptor-CC chemokine receptor 5 (CCR5). This suggests that CCR5 is involved in ALI lung damage [85]. Clinical studies also revealed that the complement system activation occurs in the lungs during ARDS, and elevated levels of C3a and C5a are observed in the serum and BALF of patients [88]. Similarly, increased MAC has been observed in the plasma of septic patients preceding the development of ARDS [89]. Elevated plasma C3a levels have also been found to be a predictive factor for patients with ARDS [90].

##### Influenza

Influenza is an acute infectious respiratory disease that occurs seasonally in temperate climates, while in tropical regions it can occur year-round, causing epidemics. It is caused by influenza viruses belonging to the *Orthomyxoviridae* family, with an RNA genome [91]. These viruses circulate in all parts of the world and cause influenza of varying severity, sometimes with hospitalization and even death [92]. Patients with severe influenza exhibit bilateral pulmonary infiltration and often die from ALI or ARDS with associated hypoxemic respiratory failure [93]. Respiratory damage, in the course of influenza, results from a combination of intrinsic viral pathogenicity and a strong host innate immune response that can exacerbate lung damage [94,95]. Therefore, the high mortality associated with the influenza virus infection may result from excessive activation of the immune system, including the complement system [96].

In the course of influenza, deposition of the complement component 3 (C3) and MAC in the lungs was observed just a few days after infection, as well as increased expression of C5aR1 in the lungs, especially on bronchial epithelial cells and inflammatory cells, and an increased concentration of C5a anaphylatoxin in plasma and BALF [97,98]. This suggests that virus-induced deregulation of the complement system activation, particularly the C5a-C5aR1 axis, is associated with systemic inflammatory responses and local tissue damage [97]. Increased levels of C5a in influenza virus infection correlated with intense infiltration of lymphocytes, neutrophils and macrophages into the airway space and with increased expression of pro-inflammatory cytokines (IL-6, TNF-α, IFN-γ, and IL-1β) [98]. A study on mice revealed that C5aR1 deficiency reduces clinical symptoms and lung damage following influenza virus infection. Histopathology showed that C5aR1-deficient mice had less interstitial inflammation and pulmonary edema, as well as reduced infiltration of lymphocytes, macrophages, and neutrophils in lung tissue. Additionally, MPO activity and intercellular adhesion molecule 1 (ICAM-1) levels were lower in C5aR1-deficient mice compared to wild-type mice [97]. Blocking the C5aR1 receptor with antibodies has similar effects. The use of anti-C5aR1 to reduce C5a-C5aR1 signaling resulted in a reduction in pneumonia and extended animal survival. There was also a reduced immune response to infection, which was characterized by lower levels of neutrophil and macrophage infiltration in the lung, and lower levels of cytokines IFN-γ, IL-1β, IL-6, TNF-α, IL-10, IL-12, IP-10, and chemokine CCL2. In addition, the use of the anti-C5aR1 antibody led to a significant reduction in the virus titer in lung tissue [97]. Similar results were obtained using the OmCI protein (a potent arthropoda-derived inhibitor of C5 activation), which binds to C5 and prevents C5a and MAC formation. When only OmCI was used, no decrease in virus titer was observed in lung tissue [98]. These results indicate that the C5a-C5aR1 axis plays an important role in the occurrence of ALI induced by influenza virus infection, and that the use of anti-C5aR1 antibodies can suppress the hyperactive immune response induced by viral infection and effectively inhibit viral replication in the lung, thereby attenuating inflammatory responses and reducing lung injury in influenza [97,98].

Infection of mice with the H5N1 virus-induced overactivation of the complement system, mainly through the lectin pathway. In lung tissues, deposition of C3, MAC, and MBL associated with serine protease-2 and increased expression of C3aR and C5aR receptors were observed [99], while, there was an increase in C3 and C5a in BALF [100]. This correlates with large numbers of alveolar epithelial cells showing degeneration and collapse, inflammatory cell infiltration accompanied by large amounts of exudates and severe edema, and exacerbation of inflammation. The use of a C3aR antagonist leads to a significant reduction in neutrophil infiltration and inflammation in the lungs, alleviating ALI and increasing survival rates. The use of a C3a receptor antagonist also reduces H5N1 virus replication in lung tissue. Similar to the C3aR antagonist treatment, anti-C5a antibody treatment reduces lung injury and neutrophil infiltration and increases survival rates. The downregulation of C3 before infecting H5N1 mice attenuated inflammatory cell injury and infiltration, reduced IFN-γ mRNA expression levels, and reduced virus titer in the lungs [99]. Other observations were made by O’Brien et al. [100]. C3 knockout (C3-/-) mice showed increased inflammatory changes along with increased macrophage infiltration. C3-/- mice showed more diffuse, non-focal pathology with increased interstitial involvement. They had moderate to moderately severe bronchiolitis and vasculitis with perivascular inflammation compared with wildtype mice, which showed mild inflammation. C3-/- mice also had significantly higher virus titer, which suggests a delay in virus clearance [100]. Infection of C3-/- mice with H1N1 influenza virus also led to the development of influenza, with a 100% mortality rate, while wildtype mice show only minor pathology and all fully recovered. C4 and FB deficient mice also showed a more severe disease course and higher mortality. Histopathological studies showed that C3-/-, C4-/- and FB-/- mice had an increased infiltration of mononuclear inflammatory cells compared to wildtype mice. In addition, multiple foci of degenerative changes were, including loss of bronchial epithelium and bronchial hypertrophy with edematous changes are observed in these mice. Higher virus titers were also found in the complement system-deficient mice. Administration of C3aR and/or C5aR antagonists to wild-type mice infected with seasonal influenza virus (H1N1) showed significant mortality (approximately 60%). Mice treated with anti-C5aR alone recovered with a mortality rate of only 10%, whereas when only anti-C3aR was administered, disease symptoms were more severe, with a mortality rate of 57%. These data suggest that signaling mediated by C3aR plays a dominant role in generating a protective immune response to pandemic influenza virus, with signaling by C5aR playing a relatively minor role [61].

In H1N1-infected lung epithelial cell cultures, FH, an inhibitor of the alternative pathway, downregulated the proinflammatory cytokines TNF-α, IL-6, CCL5, IL-12, and inhibited influenza virus entry into cells. Moreover, the C4b binding protein (C4BP) downregulated the expression of IFN-α, and NF-κB and inhibited H1N1 subtype infection in a lung epithelial cell line. In addition, similar to FH, C4BP reduces viral entry into cells [101]. The opposite effects of FH and C4BP have been observed in cell cultures infected with the H3N2 virus [101,102]. Studies on highly pathogenic influenza viruses demonstrated that these viruses cause activation or even overactivation of the complement system, which leads to inflammatory cell infiltration, exacerbation of inflammation in the lungs, and ALI. The inhibition of the complement system activation can significantly reduce inflammatory responses and attenuate ALI. Data suggest that inhibition of the complement system, or the complement system activation products, is an alternative and supportive therapeutic option for the treatment of ALI induced by infection with highly pathogenic viruses, including the H5N1 virus [97,98,99]. However, studies on C3-knock out mice infected with seasonal influenza virus show that C3 is required for protection against influenza infection, for proper virus clearance, and is associated with changes in cellular infiltration [61,100]. These results suggest that although the complement system activation levels may vary depending on the influenza virus subtype, the complement system is an important host defense mechanism against this disease, but its overactivation can lead to severe complications such as lung failure and death.

##### Severe Acute Respiratory Syndrome (SARS)

Severe acute respiratory syndrome (SARS) is a viral respiratory disease caused by SARS coronavirus, a member of the *Coronaviridae* family with an RNA genome [103]. It is characterized by severe symptoms of lower respiratory tract infection, causing alveolar damage. Atypical pneumonia with rapid deterioration and failure due to increased levels of activated proinflammatory chemokines and cytokines may occur [104]. In severe cases of SARS, ARDS is observed as a severe life-threatening immune-mediated disease.

Gralinski et al. [105], using SARS-CoV dapted to mice (SARS-CoV MA15), investigated the role of the complement system during SARS-CoV infection.They observed a significant increase in C4b, C3, and FB in the lungs of mice infected with a lethal dose of the virus compared to controls. C3 activation products (C3a, C3b, iC3b, C3dg, and C3c fragments) were detected in the lung tissue of SARS-CoV MA15-infected mice but not in control mice. C3-deficient mice (C3-/-mice) were protected from SARS, but the absence of symptoms was not associated with viral replication efficiency, and complement system activation did not result in SARS-CoV MA15 neutralization in the lung. Later in the infection, C3-/- mice exhibited reduced inflammatory monocyte and neutrophil infiltration in lung tissue, reduced T-lymphocyte activation, and less edema and lung inflammation, which correlated with improved respiratory function. In addition, silencing of C3 resulted in the absence of deposition of complement components in lung tissue, in contrast to wildtype mice. This suggests that local tissue damage by the complement system may contribute to the pathogenesis of SARS-CoV. In response to infection, an increase in the expression of MIP1a, MIP1b, and MCP-1 proteins is observed in the lung regardless of the presence or absence of C3, indicating that some inflammatory signals remain intact in the absence of the complement component. However, silencing of C3 reduced the production of G-CSF, IL-6, TNF-α, and IL-1a. All of these cytokines play significant roles in neutrophil production, recruitment, or differentiation. The absence of complement system signaling resulted in reduced SARS-CoV MA15 pathogenesis, a partial reduction in respiratory dysfunction, pathology, immune infiltration, and cytokine responses in the lung. Increased levels of C3a-derived fragments were recorded in the serum of SARS-CoV MA15-infected mice, regardless of genetic background, indicating systemic complement system activation. Additionally, elevated levels of CCL2 and CCL5 were observed. However, numerous cytokines and chemokines such as IL-5, G-CSF, and C-X-C motif chemokine ligand 1 (CXCL1) were present in much higher amounts in the lungs of wildtype mice than in C3 knockout mice. This study highlights the complement system as an important mediator of SARS-CoV-induced disease and suggests that the complement system activation regulates the systemic proinflammatory response to infection by this virus [105]. Furthermore, inhibition of the complement system signaling after SARS-CoV infection may have a role in reducing lung injury during SARS [105].

SARS-CoV infection may also be affected by the complement system activation through the lectin pathway via MBL [106]. MBL, a key molecule in innate immunity, acts as an anti-antibody prior to a specific antibody response, and the distribution of MBL gene polymorphism may influence infection. Genotypes associated with low serum MBL levels with mutated B allele are significantly more frequent in SARS patients. Moreover, the frequency of MBL haplotypes differs between SARS patients and control subjects, where the frequency of MBL deficiency-related haplotype (YB) is significantly higher. This genotype reflects the MBL levels in the serum of infected patients. The median level of MBL in these patients was significantly lower than in the control group. In contrast, carrying the mutant B allele does not influence survival in SARS-CoV infected patients [106]. Moreover, polymorphism at codon 54 (G-->A) significantly affects the susceptibility to SARS-CoV infection. This alteration may result in the reduced expression of functional MBL and thus its reduced ability to bind the pathogen and activate the complement system [107,108]. MBL can bind SARS-CoV in a dose- and calcium-dependent manner and mannan inhibiting manner in vitro, suggesting that binding occurs through MBL carbohydrate recognition domains. Furthermore, MBL enhances C4 deposition on SARS-CoV virions and has an inhibitory effect on cell infection with the virus. These studies suggest that MBL contributes to host defense against SARS-CoV and that its deficiency may be a susceptibility factor for SARS [106]. However, a study by Yuan et al. [109] found no significant differences in MBL genotypes and allele frequencies among SARS patients and controls. On the other hand, they showed that polymorphisms of human Fc γ receptor IIA (FcγRIIA) genes influence the infection and course of SARS. The FcγRIIA-R/R131 genotype was associated with a more severe course of SARS; its homozygosity was more frequent in SARS patients requiring intensive care unit treatment than in controls [109].

##### Middle East Respiratory Syndrome (MERS)

Middle East Respiratory Syndrome (MERS) is a viral infectious disease with a high mortality rate of up to 36%. The etiological agent of the disease is MERS-CoV of the *Coronaviridae* family [103], which is a zoonotic virus, meaning that most cases of infection are transmitted from animals to humans [110]. MERS-CoV is similar to SARS-CoV, which belongs to the same genus of β-coronaviruses, and caused an outbreak in 2002–2003 with a death rate of 9.6% [110]. MERS is characterized by progressive severe pneumonia with diffuse alveolar damage in the acute phase [111]. Studies indicate that MERS-CoV induces a cytopathic effect and disrupts the host immune response, similar to SARS-CoV [112,113]. The high pathogenicity accompanied by increasing dysregulation of immune responses draws attention to the possible involvement of the complement system in the pathogenesis of MERS.

MERS-CoV infection increases MAC deposition in lung tissue [114] and elevates the expression of C5aR in the lung, especially in bronchial epithelial cells, pneumocytes, and inflammatory cells. Increased levels of C5a in the serum of virus-infected patients can also be observed. It is speculated that MERS-CoV can rapidly induce activation of both local and systemic activity the complement system [114]. Increased C5a levels can lead to mast cell degranulation, chemotaxis of pro-inflammatory cells, production of cytotoxic oxygen radicals, which leads to increased systemic inflammatory response and local lung tissue damage [115]. MERS-CoV infection leading to complement system overactivation may contribute to pyroptosis (a highly inflammatory form of lytic programmed cell death) and inflammation in human macrophages [116,117]. It has been observed that MERS-CoV infected macrophages induce the production of pro-inflammatory cytokines and chemokines [113]. In cell cultures of THP-1 monocytes and differentiated THP-1 macrophages, MERS-CoV induces caspase-1 and pro-IL-1β activation. In contrast, only THP-1 macrophages have shown increased levels of caspase-1 protein, pro-IL-1β, and the active form of IL-1β [116]. MERS-CoV infection also increases complement system expression. There was increased expression of C3 and the C3aR receptor in both monocytes and macrophages. There was also an increase in the expression of C5aR1, which when combined with C5a has a pro-inflammatory effect. Interestingly, MERS-CoV infection decreased the expression of the C5aR2 receptor, which is attributed to anti-inflammatory activity [116]. The studies on mice demonstrate that blocking the C5a-C5aR pathway by anti-C5aR antibodies reduces the inflow of macrophages to the lung tissue. Expression of the IFN-γ receptor, located mainly on inflammatory cells, was also reduced. Additionally, inhibition of C5aR signaling leads to a decrease in proinflammatory cytokines (IL-1β, TNF-α, IFN-γ, and IL-12) and chemokines CCL2, but does not affect the level of proinflammatory IL-6 [114,116]. These observations suggest that silencing of the C5a-C5aR pathway may reduce local and systemic inflammation, especially the Th lymphocyte response that is induced by MERS-CoV infection [114]. The use of anti-C5aR antibodies reduces lung inflammation and infiltration of inflammatory cells (lymphocytes, neutrophils, and macrophages).

MERS-CoV infection also causes damage to the spleen, which is an important peripheral lymphoid organ, and can produce an immune response immediately after viral infection, and lymphopenia is the primary clinical aspect of severely ill patients [112]. In the histopathological picture, necrotic and apoptotic changes are observed especially in the white pulp and inflammatory infiltrates in the red pulp. However, studies suggest that its damage is not related to the virus replication itself in splenocytes, but to a dysregulated immune and inflammatory response caused by MERS-CoV infection in the lung and overactivation of the complement system. Inhibition of the receptor for C5a reduces splenic damage (reduced cell necrosis and apoptosis), decreases the expression levels of caspase-1, IL-1β, and pyroptosis, and increases the number of macrophages in the red pulp and even affects its regenerative processes [114,116]. In contrast to the lack of effect of C3 silencing on SARS-CoV replication [105], Jiang et al. [114] demonstrated that the use of anti-C5aR antibody reduced the expression of viral antigen in lung tissue. In addition, reduced viral replication and lower virus titer were found in the lung tissue of mice treated with anti-C5aR Ab. This indicates that inhibition of the complement system activation may reduce MERS-CoV replication in the lung, suppress excessive activation of the inflammatory response, and reduce organ damage in MERS.

##### Coronavirus Disease 2019 (COVID-19)

Coronavirus disease 2019 (COVID-19) is an acute respiratory infectious disease caused by the SARS-CoV-2 virus, which, like the previous two viruses, belongs to the *Coronaviridae* family [103]. Most cases of COVID-19 have a benign course, but some can lead to life-threatening pneumonia and multiple organ failure (MOF) [118]. In the latter cases, increasing evidence suggests that SARS-CoV-2 is not always confined to the respiratory tract but can also spread to other organs. Most patients with COVID-19 also have symptoms other than respiratory disorders, including neurological, cardiovascular, intestinal, and renal disorders [119]. SARS-CoV-2 infection triggers activation of the innate immune system, and its dysregulation and overactivation, in which the complement system plays a key role, can lead to ARDS and significant cytokine release [120,121]. Patients hospitalized with COVID-19 have consistently elevated systemic complement system activation (MAC, C5a, C3bc, C3bBbP, and C4d). However, patients who developed respiratory failure exhibit only elevated levels of MAC and C4d [122]. MAC deposition can affect endothelial leukocyte migration, intra-alveolar endothelial damage, and microvascular damage and vascular leakage leading to respiratory failure [123]. None of the other complement system activation products is significantly associated with changes in parameters characterizing ARDS in COVID-19. Components of the complement system also correlated with markers of systemic inflammation. Ferritin, C-reactive protein (CRP), and white blood cell counts are positively correlated with MAC levels, whereas C4d and C3bc correlate only with ferritin levels [122]. Elevated plasma levels of MAC and C5a have also been observed in patients with moderate to severe COVID-19 compared to healthy subjects [124,125]. MAC levels are significantly higher in patients with severe symptoms compared to moderate ones, while no difference in C5a levels has been observed between these patient groups [124]. Other studies [125,126] show that C5a levels are significantly higher in COVID-19 patients requiring intensive care unit treatment, and the highest C5a levels have been demonstrated in patients requiring mechanical ventilation.

Carvelli et al. [127] demonstrate that the elevated C5a levels observed in COVID-19 patients may be related to leukocyte infiltration into the lungs and subsequent lung dysfunction. In addition, anaphylatoxin C5a may be related to the cytokine storm observed in ARDS during COVID-19. Increased expression of C5aR1 on peripherally circulating neutrophils and monocytes has also been observed in SARS-CoV-2 infected individuals. Consistent with the inflammatory function of C5a and the expression of C5aR1 on monocytes, C5a increases the production of the inflammatory cytokines IL-6, TNFα, and CCL2. C5a has also been detected in BALF from COVID-19 patients with ARDS. Its increased levels are associated with increased levels of proinflammatory cytokines (CXCL8, CXCL9, CCL2, CCL4, IL-6, TNF-α, and IL-1β) in BALF. Additionally, BALF has increased numbers of neutrophils and monocytes with increased expression of C5aR1 and elevated levels of transcripts for inflammatory cytokine genes (CXCL8, CCL2, CCL4, CXCL9, TNF-α, and IL-6). These data indicate that C5a production leads to chemotaxis and cell activation in the lung and contributes to the release of inflammatory cytokines. Multiple immunohistochemical analyses of lung tissue from patients who died from COVID-19 confirm macrophage infiltration, a significant proportion of which have increased expression of the C5aR1 receptor with pro-inflammatory functions [127]. In lung tissues, extensive deposition of C1q, C3, C4, and MAC has also been observed in the capillaries of the alveolar septum and, to a lesser extent, on alveolar cells [123,126,128]. These findings suggest that the classical pathway of the complement system is a common pathway for its activation in the lungs of patients with COVID-19 [128].

Deposition of FB has also been discovered in lung tissue samples from COVID-19 patients [128]. Elevated levels of FB and an elevated C3b/C3 ratio are also observed in the plasma of COVID-19 patients requiring treatment in the intensive care unit and/or mechanical ventilation [125]. These observations indicate that in addition to the classical pathway, the alternative pathway must play an important role in the complement system activation in COVID-19. The levels of FD, which induces the breakdown of FB and influences the activation of the alternative pathway, are significantly higher in patients who died of COVID-19. Moreover, FD correlates strongly with markers of endothelial damage (angiopoietin-2) and prothrombotic status (thrombomodulin and von Willebrand factor antigen) [125]. Researchers also point to the involvement of the C5 component in thrombosis. It can increase the expression of functionally active tissue factor (TF) in leukocytes and endothelial cells [129]. Therefore, it is noted that the activation of the complement system is mainly associated with multiorgan failure in COVID-19 due to its role in endothelial damage and its induction of a prothrombotic state [125].

Researchers have also highlighted the role of the lectin pathway of the complement system activation in COVID-19 [122,126,128,130]. Increased levels of MBL in lung tissue have been observed in patients infected with SARS-CoV-2 [122,126]. This increase is consistent with intense tissue expression of IL-6, TNF-α, and intercellular adhesion molecule-1 (ICAM-1) which may lead to inflammation-induced endothelial activation and increased likelihood of thrombus formation [130]. In addition, it has been shown that MBL correlates with the level of acute-phase CRP, which indicates that the lectin pathway of the complement system activation is related to inflammation [122]. The activation of the lectin pathway may occur through the interaction of SARS-CoV-2 protein N with MASP-2. Protein N may regulate the dimerization, activation and cleavage of MASP-2 and its binding to MBL. In addition, protein N affects C4 cleavage and enhances C4b deposition. Along with C4b deposition, deposition of activated C3 is markedly increased which suggests increased C3 convertase activity. In addition, coronavirus N protein increases the deposition of the MAC complex. These findings indicate that the SARS-CoV-2 protein N effectively promotes the complement system activation and opsonization by its components [126]. Intense activation of the lectin pathway, with particular emphasis on the MBL pathway, together with endothelial dysfunction and massive production of proinflammatory cytokines, may lead to a poorer prognosis in patients infected with SARS-CoV-2 [126,130].

Different observations were made by Macor et al. [128] who did not detect tissue deposits of MBL in the lung tissue of patients who died from COVID-19 except for occasional deposition of this protein in vascular thrombi. MASP-2, which is essential for the activation of the lectin pathway, was also not detected [128]. On the other hand, the authors of [131] performed an exploratory study indicating that therapeutic complement inhibition (C3 and C5 components) via AMY-101 and ekulizumab abrogates COVID-19 hyperinflammation. The study was a comparative assessment of key clinical and biochemical correlates in two small COVID-19 patient cohorts with SARS-CoV-2 associated ARDS treated either with the C3-based therapeutic AMY-101 and with the C5-targeting mAb eculizumab. Eculizumab is a clinically approved anti-C5 mAb that targets exclusively the terminal pathway whereas AMY-101 is a C3-targeted drug candidate based on third-generation compstatins. Administration of eculizumab and AMY-101 elicited a robust anti-inflammatory response, reflected by a steep decline in C-reactive protein and IL-6 levels.

Interestingly, the administration of inhibitors to patients influenced a rapid and steady decrease in the number of neutrophils in the blood and effectively reversed COVID-19-associated lymphopenia in severe forms of the disease. The inhibition of C3 and C5 also increased platelet counts in patients with thrombocytopenia induced by COVID-19 and reduced the incidence of thrombosis. Treatment decreased C3a levels in COVID-19 patients, inhibited the alternative pathway, and reduced MAC levels. The potent anti-inflammatory profile and the effects of both complement system inhibitors on the markers of COVID-19 coagulopathy were reflected in a marked improvement in pulmonary respiratory function in all non-intubated patients. This improvement culminated in the complete resolution of ARDS and relief of bilateral interstitial pneumonia associated with SARS-CoV-2 [131]. These findings suggest that the complement system is systemically activated in the majority of cases with COVID-19, while its activation may contribute to the development of lung and endothelial damage in COVID-19, as well as lead to failure of other organs such as heart and kidney or even to MOF.

#### 2.1.2. Viral Lower Respiratory Tract Illness (VLRTI)

Respiratory syncytial virus (RSV) is the most important cause of viral lower respiratory tract illness (VLRTI) in infants, children, the elderly and immunocompromised patients. The virus belongs to the *Pneumoviridae* family, with a RNA genome [132]. The name of the virus is related to the fact that during the replication of the virus, neighboring cells come together to form large multinucleated complexes [132]. Clinical manifestations of the disease range from asymptomatic infection to a form with bronchospasm and pneumonia [133]. In mice, acute RSV infection causes airway hyperresponsiveness (AHR), inflammation and excessive mucus secretion, and inflammatory cell influx into the lungs [134]. Infected cells induce the complement system activation, producing the anaphylatoxin C3a, and the deposition of IgG-C3 complexes can be observed in lung tissue [134,135]. Subsequently, C3a-C3aR interactions induce tachykinin production, followed by the development of AHR, inflammation, and excessive mucus secretion [134]. In contrast, targeted deletion of the C3a receptor gene (C3aR1-/-) protected mice from developing AHR and induced reduced neutrophil infiltration. C3aR-/- mice showed significantly reduced expression levels of the gene encoding the mucus-associated protein gob5 compared with wildtype mice. These facts suggest a possible role for the C3a–C3aR interaction in maintaining basal levels of mucus production in the absence of airway inflammation. It has also been shown that silencing the receptor for C3a also increased the rate of RSV clearance from cells [134]. In contrast, silencing C3aR did not reduce the levels of the cytokines TNF-α, IFN-γ, IL-4, and IL-5, while it significantly reduced the levels of IL-6 and IL-17A, which is implicated in the production of AHR in acute RSV infection [134]. C3-deficient mice showed significantly better lung function parameters compared to wildtype mice [135]. The same study showed that children with acute VLRTI had the deposition of peribronchial C4d, which provided evidence for activation of the classical complement system cascade [135]. The complement system factors may also promote T-lymphocyte activation, which is the case in enhanced respiratory syncytial virus disease (EDR) [135]. In addition, EDR is characterized by bronchospasm and pneumonia [136], both of which may be related to anaphylotoxin activity [137,138]. Furthermore, RSV infection has been shown to induce a mixed Th1 and Th2 response, with an increase in IFN-γ and IL-10 [139,140].

### 2.2. The Complement System in Acute Liver Failure (ALF)

Acute liver failure (ALF) or fulminant hepatic failure (FHF) is a disease with several etiologies. The most common causes of ALF are drug-induced liver damage, viral diseases, e.g., HBV and autoimmune hepatitis [141]. This disease is characterized by massive necrosis of hepatocytes, rapid deterioration of liver function, encephalopathy or coma, and high mortality. In addition to the direct action of hepatotoxic agents, various immune cells are activated and recruited to the liver [142]. They produce numerous proinflammatory mediators including cytokines, chemokines, proteases, and reactive oxygen species, inducing apoptotic or necrotic changes in hepatocytes [143]. The complement system is also involved in the pathogenesis of ALF. Kusakabe et al. [144] investigated the potential therapeutic role of C5 inhibition in ALF in a mouse model. They induced ALF in wildtype and C5-deficient mice and used the anti-C5 antibody as a treatment option. C5 deficiency significantly reduced the influx of macrophages and neutrophils into the liver, liver damage, and extended the length of survival. Additionally, it affected the decreased expression of proinflammatory cytokines (TNF-α, IL-6, and IL-1β) and chemokines (CXCL1 and CXCL2). However, just the decrease in mRNA expression for TNF-α correlated with decreased serum levels of TNF-α protein. Moreover the C5aR antagonist significantly decreased TNF-α production by activated macrophages [144], which are one of the major extrahepatic sources of complement proteins [145]. These observations suggest a key role for anaphylatoxin C5a in ALF-mediated liver damage mediated by TNF-α and macrophages [144].

Increased deposition of C3a, C5a, and MAC was also observed in liver tissues of ALF patients [146]. A dramatic increase in serum C5a was observed in mice in which ALF was induced by D-GalN/LPS. In addition, there was a significant increase in mRNA expression and protein levels for C5aR in liver tissue compared to the control group. In contrast, a blockade of C5aR signaling decreased serum ALT levels, pro-inflammatory cytokine levels (TNF-α, IL-1β, and IL-6), reduced liver tissue damage, and increased survival rates. These parameters indicate that C5aR blockade affects ALF attenuation [146,147]. Another factor involved in the pathogenesis of ALF appears to be sphingosine kinase 1 (SphK1), which through sphingosine-1-phosphate synthesis and NF-κB activation promotes the inflammatory response in ALF. The use of anti-C5aR antibodies resulted in a significant decrease in SphK1 expression in liver tissue and peripheral blood mononuclear cells (PBMCs) after ALF induction [146]. Similar to ALF patients, C3 deposition in liver parenchyma was observed in ALF-induced mice. In addition, C3aR and C5aR receptors can be observed not only on non-parenchymal cells, especially Kupffer cells, but also on hepatocytes. The mRNA levels of anaphylatoxin receptors C3a and C5a correlate with immunohistological data which show that ALF is followed by the complement system activation, and the degree of the complement system activation correlates with the severity of the liver injury. In contrast, studies in C3-deficient (C3-/-) mice have shown that this component played a key role in liver damage during ALF. C3-/- mice after D-GalN/LPS treatment exhibited a significantly lower extent of liver damage than wild-type mice. In addition, silencing of C3 resulted in a significantly lower extent of hepatocyte damage, and less inflammatory cell infiltration. Reduced MAC deposition in liver tissue and decreased serum levels of the cytokines TNF-α and IL-6 were also observed. Similar to C3 silencing, anti-C3aR treatment significantly reduced hepatic hemorrhage, parenchymal damage, and decreased ALT and proinflammatory cytokine levels in serum [147]. These studies suggest that ALF induction is followed by overactivation of the complement system, and that C5a/C5aR and C3a/C3aR signaling play key roles in the pathogenesis of ALF.

#### Hepatitis B (HB)

ALF caused by hepatitis B virus (HBV), is rare (about 1% of patients hospitalized for acute hepatitis B (HB)) and is one of the most dangerous human infectious diseases with high mortality [148,149,150]. Hepatitis B is a major global health problem. The inflammatory response mediated by the immune system is the major cause of HBV-related liver damage [151]. Gene ontology (GO) analysis showed decreased expression of complement system genes (MBL2, MASP2, C9, FB, C6, C5, and C8) in HBV-induced ALF. However, analyses of protein–protein interactions (ppi) revealed that some of the most important node-degree genes affecting the expression of other genes in ALF include complement system component C5 and FD. The levels of these genes in the HBV-related ALF group were significantly higher than those in the control group [148]. Chen et al. [152] demonstrated that in HBV-induced ALF, there is an increased ability of C1q to bind to immune complexes. This results in increased deposition of complement system products in the liver and increased activation of the classical pathway, leading to increased lysis of infected hepatocytes. In addition, extensive intrahepatic infiltration of CD20+ cells and plasma cells producing IgM and IgG is observed. The intrahepatic expression of immunoglobulins is accompanied by deposition of complement system component C1q [152]. Therefore, researchers suggest that the complement system may be essential in the pathogenesis of HB-ALF, and recognition of HBV antigens on the cell surface by high-affinity intrahepatic antibodies may lead to extensive complement system-dependent cytotoxicity and liver damage [148,152,153,154]. This mechanism is consistent with the dramatic clinical course of ALF, where massive hepatic necrosis can occur within hours of disease onset [148,152]. However, reduced C1q levels are observed in the plasma of patients with HBV-induced acute-on-chronic liver failure (ACLF) (HBV-ACLF). In contrast, the study showed no change in MBL and FB levels compared to the control group. Similar to C1q, decreased plasma levels of C3 and C4, their degradation products (C3a and C4a), and MAC have been observed in the HBV-ACLF group, which may be related to the increased accumulation of these components in the liver of ACLF patients [152]. Li et al. [155], after examining the levels of C3 degradation products in plasma samples, observed increased levels of iC3b (which are C3 cleavage fragments) and increased levels of anaphylatoxin C3a in HBV-ACLF patients compared to the chronic hepatitis B group. These observations indicate increased activation of the complement system in the acute course of HB [155]. It has also been shown [156] that stimulation with HBV antigen (HBsAg) in the presence of C5a or C5a/C3a modulates cellular immune responses and affects the increased production of IL-2 and IFNγ in the whole blood collected from healthy individuals. Anaphylatoxins during HBV infection may also affect antigen-presenting cells. Increased expression levels of MHC class II and CD86 are observed after the addition of C3a and C5a. Innate signals mediated by complement system pathways contribute to HBV-specific cellular immune responses. However, its overactivation may lead to exacerbation of inflammation through increased production of pro-inflammatory cytokines [156].

HBV also interacts with the CD59 protein, which is responsible for MAC inhibition. Studies on human hepatocytes (HepG2, BEL7402, and HL7702) transfected with HBV core antigen (HBc) and on HBV-infected mice [151,157] show a significantly reduced level of CD59 protein on the cell surface [151]. The decreased amount of protein is accompanied by a decrease in its expression at the mRNA level [151,157]. The decreased level of CD59 expression results in increased sensitivity of HBc-transfected hepatocytes to complement system-mediated lysis [8]. These observations are confirmed by immunohistochemical and PCR studies of liver biopsies from HBV-infected patients [151,157]. MAC and HBc are accumulated on the hepatocytes of patients but not on the hepatocytes of healthy individuals. Moreover, the amount of MAC and HBc deposited increases with the severity of liver failure, while the expression level of CD59 decreases. The levels of C3 and C4 in peripheral blood have also been measured. Compared with healthy references, the amount of complement system C4 is significantly reduced in HBV-infected patients, while no change was observed in C3 levels, indicating that more C4 is consumed during MAC formation [151]. Decreased CD59 expression and increased MAC formation and deposition elevate sensitivity to the complement-dependent cytotoxicity and promote cell lysis, thereby exacerbating damage and stimulating liver necrosis in HB [151,157].

### 2.3. The Crosstalk between the Complement System and Coagulation

Disseminated intravascular coagulation (DIC) syndrome is observed during viral diseases with hemorrhagic fever. It is characterized by systemic activation of blood coagulation resulting in fibrin formation and deposition, leading to microvascular clots in various organs and contributing to multiple organ dysfunction syndrome (MODS) [158,159]. DIC can also lead to massive life-threatening hemorrhages due to the consumption of clotting factors and platelets [160]. The functions of the complement system and coagulation pathways in acute conditions are closely related [161]. Activation of the complement system and coagulation leads to conversion of zymogens and assembly of proteolytic complexes, which are mostly serine proteases [162]. The relationship between coagulation and the complement system occurs in both directions. The complement system proteins can activate the coagulation cascade, but also some coagulation enzymes, such as thrombin and factor Xa, can directly activate components of the complement system cascade, while the thrombomodulin-protein C anticoagulation pathway can inhibit the complement system activation [86,163]. In addition, coagulation factor XIIa acts in both coagulation contact activation and the complement system activation through the classical pathway, and C1 inhibitor, in addition to its role in the complement system, is also a potent neutralizer of coagulation factor XIa [164,165]. Therefore, severe trauma and acute blood loss are associated not only with DIC but also with massive complement system activation. This results in the production of potent anaphylatoxins C3a and C5a, which in turn can enhance coagulation [166,167,168]. Anaphylatoxin C5a promotes procoagulant activity by upregulating TF expression by endothelial cells and neutrophils and changing mast cell and basophil activity from profibrinolytic to prothrombotic by increasing plasminogen activator inhibitor-1 activity [169,170,171]. Activated platelets can activate the complement system via classical and alternative pathways, while MASP-2 involved in the activation of the lectin pathway is able to produce thrombin by direct cleavage of prothrombin [172,173]. MAC also affects the coagulation system. It has similar activity towards prothrombin as factor V and exhibits procoagulant activity mediated by the induction of TF expression by endothelial cells [174,175]. The coagulation and complement systems are closely related and are mutually regulated to achieve effective host protection. However, uncontrolled activation of these enzyme cascades has a major impact on organ failure and patient death [176].

#### 2.3.1. Ebola Virus Disease (EVD)

Ebola virus disease (EVD) is one of the most dangerous infections in the world. It is a severe and often fatal disease caused by the Ebola virus (EBOV), a member of the *Filoviridae* family of RNA genomes [177,178]. EVD has a high case fatality rate (mean 50%) and is characterized by fever and gastrointestinal symptoms [177]. Initially, patients have non-specific flu-like symptoms and eventually progress to DIC, shock, and MOF [179]. EBOV tends to infect various immune cells (dendritic cells, monocytes, and macrophages), endothelial and epithelial cells, hepatocytes, and fibroblasts, where it actively replicates through gene modulation and apoptosis and exhibits high viral load [180]. The virus reaches regional lymph nodes, causing lymphadenopathy and blood-borne dissemination to the liver and spleen, promoting an active inflammatory response [181]. The production of cytokines and chemokines causes a dysregulation of the immune response by disrupting the harmony of the vascular system, ultimately causing DIC and multi-organ dysfunction [182]. The complement system as a component of immunity appears to be an important element in EBOV infection and in EVD.

Recombinant human MBL (rhMBL) has been shown to effectively inhibit infection of human cells by EBOV in the presence of active serum complement system [183,184]. MBL binds to invariant glycans on EBOV virus, enhances immunophagocytosis, activates the lectin pathway of the complement system, enhances the host response in cooperation with TLR2/6, and regulates cytokine production [51,52]. MBL also blocks the interaction of EBOV glycoprotein with DC-SIGN dendritic cell lectin, with the strength of virus neutralization depending on the concentration of complement components and activation [52]. Researchers [51] have observed a paradoxical MBL-dependent increase in EBOV infection in primary human macrophage cell lines and human monocyte-derived macrophage cell lines [51]. However, this effect occurred only when other complement components were at low concentrations. EBOV protects itself from the host response by down-regulating host proteins, including immune regulatory molecules and receptors such as major histocompatibility complex class I (MHC1) and β1 integrin [185,186]. It can also inhibit the complement system activation and cause a downregulation of its components. They found that although the main site of EBOV infection is the liver, the expression of the complement system genes was impaired in many tissues, suggesting a systemic response. Jayaprakash et al. [187] observed that there is elevated expression of C3P1, C4b, C5, C9, C6, and MASP1 during EBOV infection, while C1R, C3, C8g, and MASP2 are down-regulated [187]. Interestingly, it has been shown that C4a deficiencies can increase serum MBL levels, and the occurrence of this imbalance may contribute to the severity of EBOV infection [51]. The results of a study by Furuyama et al. [188] will reveal that the interaction of EBOV-antibody-C1q complexes with C1q receptors on the cell surface leads to an increased viral entry into cells. These observations have been confirmed by research using the anti-C1qR antibody. Anti-C1qR significantly reduces the antibody-dependent enhancement (ADE) mechanism, and viral entry most likely, by blocking the interaction between C1q and gC1qR. Since C1q is present in plasma at a relatively high concentration and C1q receptors are expressed in many cell types (including endothelial cells), it is possible that EBOV infection of endothelial cells may be caused by C1q-mediated ADE at a late stage of the disease and may contribute to the exacerbation of hemorrhagic symptoms [188].

#### 2.3.2. Dengue

Dengue is a viral disease transmitted by mosquitoes in tropical parts of the world. The incidence of dengue has grown dramatically around the world in recent decades [189]. It is caused by four different serotypes of dengue virus (DENV 1-4) [190], a member of the *Flaviviridae* family, with an RNA genome [191,192]. The clinical manifestations of dengue can vary in terms of severity: from the so-called classical form of dengue fever (DF), a fever lasting about 4–7 days; to the life-threatening dengue hemorrhagic fever (DHF) and dengue shock syndrome (DSS) [189]. DHF and DSS are characterized by increased vascular permeability and plasma leakage, respiratory failure, severe bleeding, and impaired organ function [189,193]. Many immunomodulatory and vasoactive factors, such as TNF-α, IL-1, IL-6, macrophage inhibitory factor, and metalloproteinases from macrophages or dendritic cells, are associated with severe dengue or DENV-induced vascular dysfunction [194,195,196]. This suggests that the pathogenesis of dengue is multifactorial and predominantly caused by a dysregulated immune response. Researchers are also increasingly focusing on the complement system as a missing piece of the puzzle in the pathogenesis of dengue [197].

The liver is one of the most damaged organs in dengue. Vacuolization of the cytoplasm and apoptotic nuclei of hepatocytes, a large infiltration of eosinophils and lymphocytes, and extensive hepatic necrosis are observed. In addition, areas of damage show the deposition of MAC, which can also be seen in Kupffer cells and hepatic macrophages, particularly in areas associated with liver damage. MAC deposits are also present in the subendothelial area of small- and medium-sized blood vessels. Despite the sporadic presence of C1q and C3b in the damaged areas of the liver, increased deposition is observed in the spleen. Moreover, MAC occurs as intense and diffuse deposits in splenic vesicles. These components correlate with immunoglobulins and with the E envelope protein of DENV [198]. It has been suggested that complement system anaphylatoxins are generated by complexation of DENV with an antibody, and their occurrence along with MAC in the circulation is correlated with clinical severity, peaking at the time of maximal vascular leakage [198,199,200,201]. C3 levels are also related to disease severity [202]. DHF patients show a decrease in C3 levels compared to dengue patients without hemorrhagic fever (DF) or controls. The decrease in C3 is associated with increased complement activity, where its cleavage is faster than synthesis. These assumptions are supported by the fact that DHF patients have elevated levels of the anaphylatoxins C3a (a product of C3 cleavage) and C5a. Decreased levels of C3 and increased levels of anaphylatoxins are correlated with the severity of dengue. Such a correlation is also shown by the levels of oligomerized MBL and C4a in the plasma of DHF patients. Their levels are also higher than in DF patients during the acute phase of infection. These observations suggest that it is possible that in DHF, MBL may contribute to the increased activation of (C4b2b) C3 convertase through mechanisms that do not involve immune complexes. Interestingly, the abnormal complement system activation in dengue may be related to an imbalance in the levels of FD and FH in DHF patients. They show elevated levels of FD which cleaves FB with the production of active C3 convertase and decreased levels of FH responsible for inactivating C3 convertase [202].

Interestingly, other researchers [197] have demonstrated that significantly increased mRNA for FH is present in endothelial cells and macrophages but does not reflect protein levels. However, surface-bound and intracellular FH protein is induced by DENV, but only in DENV-positive antigenic cells, whereas in two other DENV-susceptible immortalized cell lines ARPE-19 and HREC, FH protein is induced both intracellularly and extracellularly by DENV infection. Elevated mRNA levels have also been observed for FB, but its levels correlated with elevated FB protein in all cell types. However, regardless of cell type, there is an imbalance in the components of the alternative pathway with lower levels of FH compared to FB and an increase in markers of alternative pathway activity in DENV infected cells. This imbalance may lead to increased deposition of complement system component C3b on the surface of DENV infected cells and with an increased ability to promote lytic activity [197]. This imbalance leads to the overactivation of the complement system, production of anaphylatoxins and exacerbation of inflammation, which consequently may lead to acute organ damage in the course of DHF [197,202]. The complement system activating factor appears to be the non-structural protein 1 (NS1) of DENV. This protein is located on the surface of infected cells and can also be released into the external environment [203]. NS1 is highly immunogenic, and specific anti-NS1 antibodies play a role in protection against disease [204,205]. High levels of NS1 are found in the bloodstream of patients in the acute phase of dengue [206].

Researchers have demonstrated [200] that NS1 is involved in the complement system activation. Soluble NS1 activated the complement system cascade until the end product MAC was produced. Higher levels of NS1 and MAC were observed in the plasma of DHF patients in the acute phase of the disease compared to DF patients. Additionally, MAC levels were strongly correlated with disease severity. Interestingly, high amounts of NS1, complement system anaphylatoxin C5a and MAC were detected in pleural fluid of patients with DSS. All values were higher than in the plasma of the same patients, indicating the accumulation of analytes probably due to local leakage. These data suggest that NS1-mediated complement system activation leads to local and systemic production of anaphylatoxins and soluble MAC, which may contribute to the pathogenesis of vascular leak-age occurring in DHF/DSS patients [200]. Another hypothesis [207] suggests that vascular leakage may be caused by selective binding of cation chemokines interacting with complement system anaphylatoxins produced locally on DENV-infected cells.

Giang et al. [208] noted the association between MBL2 polymorphisms (one of the two genes encoding MBL responsible for activation of the complement system via the lectin pathway) and DENV infection. They associated an increased risk of dengue fever with part of the 550H promoter allele and the HXPA haplotype with a protective role against DENV infection, and the XO and LXPB haplotypes. They also observed elevated levels of C2, C5, and C5a in patients with more severe dengue with a concomitant reduction in serum FD levels [208]. MBL appears to be of particular importance in neutralizing the virus and inhibiting the course of dengue. MBL binds to all serotypes of the virus. Patients with higher blood MBL concentrations had stronger neutralization of DENV [209]. MBL-dependent neutralization occurs partly by blocking viral fusion with cells and is partly dependent on C3 and C4. Experiments in mice showed MBL-dependent accelerated virus clearance [56]. All the data presented indicate the involvement and role of the complement system in the infection and course of DF and DHF. The complemnt system may play a positive role by neutralizing the virus, decreasing its titer, and alleviating the infection. On the other hand, imbalance in its components or excessive activation may lead to exacerbation of the disease and severe complications in the form of vascular hyperplasia and massive hemorrhage, which may result in acute organ failure.

### 2.4. The Complement System in Vector-Borne Diseases (VBDs)

Vector-borne diseases (VBDs) are illnesses caused by viruses, parasites, or bacteria that are transmitted by a vector such as mosquitoes, ticks, sandflies, triatomine bugs, tsetse flies, fleas, black flies, aquatic snails, and lice [210]. VBDs account for more than 17% of all infectious diseases, causing more than 700,000 deaths annually [210].

#### 2.4.1. Zika Virus Disease (ZVD)

Zika virus disease (ZVD) is caused by a virus transmitted primarily by *Aedes* mosquitoes, which bite during the day. Zika virus (ZIKV) is a member of the *Flaviviridae* family [192]. ZIKV infection often goes unnoticed or is asymptomatic in about 80% of cases. Initial infection most likely occurs in human skin cells directly affecting permissive human skin fibroblasts, epidermal keratinocytes, and immature dendritic cells. However, in some cases, ZIKV infection can lead to severe thrombocytopenia and profuse bleeding and nervous system damage [211]. The classical complement system pathway has been identified as a major factor for the complement system activation in ZIKV infection [212,213]. It results in the neutralization and reduction of virus titers in the body during infection. Studies show that natural IgM antibodies in human serum are involved in complement-mediated neutralization of ZIKV. This may be due to the deposition of proteins such as C3b fragments (which may hide viral epitopes important for infection) or by lysis of virions due to MAC formation. However, studies have shown that a decrease in viral RNA copy number, and thus a decrease in viral titer, only occurred when MAC was formed. Therefore, MAC-mediated viral lysis, rather than opsonization, appears to be essential for the reduction in viral titer during in ZVD [213].

ZIKV activates the classical complement system pathway and therefore must have evolved escape mechanisms that limit virolysis. The ZIKV E protein (ZIKV E) binds to components (C7, C8, C9, and C5b6) of the terminal pathway complement. Further analyses revealed that ZIKV E protein interfered with the polymerization of C9, induced on cellular surfaces, either by purified terminal complement proteins or by normal human serum (NHS) as a source of the complement. This data indicates that ZIKV reduces MAC formation and complement-mediated lysis by binding terminal complement proteins to the viral E protein [212]. In addition, ZIKV E bind to C1q, which is an important initiator of the complement system activation in the classical pathway and affected C1q inhibition [212,213]. Another mechanism of complement system evasion by ZIKV is the incorporation of the regulatory protein CD55 into the viral envelope, which contributes to virus stability and helps avoid complement-dependent virolysis [70]. The complement system may be involved in the pathophysiology of brain damage in ZIKV infection [214]. Animals infected with ZIKV develop antibodies to C1q, which may contribute to neurological complications in the course of ZVD. Infection with cross-reacting anti-C1q antibodies may not only affect the acute course of the disease but also impair normal brain development and function long after ZVD has resolved [214]. Interestingly, despite the ability of ZIKV to bind C1q, the brains of ZIKV-infected mice showed increased expression of complement proteins, C1q and C3 [215]. This may be because anti-C1q antibodies or ZIKV infection may activate complement system components and contribute to neurological diseases and thrombocytopenia [214]. In addition, increased expression of complement system components and elevated TNF-α levels promote exacerbation of infection. In the case of ZIKV, this leads to engulfment of presynaptic terminals by microglia and their damage and cognitive deficits [215].

#### 2.4.2. West Nile Fever (WNF)

West Nile fever (WNF) is a disease caused by the West Nile virus (WNV), which belongs to the *Flavivididae* family of RNA genomes [192]. WNV is a neurotropic virus and can cause encephalitis. In most cases, the virus is transmitted by mosquitoes of the genus *Culex*, but transmission can also occur by blood transfusion, organ transplantation, breastfeeding, or intrauterine exposure. Infections in humans are mainly subclinical, but reported signs of infection can include fever and myalgia, meningitis, and death [216]. There are also cases of WNV infection with diffuse hemorrhagic manifestations [217]. An efficient host immune system response is required to protect against lethal infection because genetic or acquired macrophage or lymphocyte deficiencies or dysregulation of the complement system activity result in a higher viral load on the central nervous system and body [218,219]. In WNV-infected mice, a decrease in C3 and C4 activity was observed along with an increase in C3dg fragments, indicating the complement system activation. Additionally, mice genetically deficient in any component of C1q, C4, FD, and FB were more susceptible to infection and showed higher mortality. This suggests that all activation pathways operate together to limit the spread of WNV [220]. Moreover, mice deficient in C3 or CR1 and CR2 receptors have shown increased central nervous system (CNS) virus load and were susceptible to lethal infection at a low dose of WNV. These mice also had significant deficits in humoral response with reduced IgM and IgG levels [221]. The researchers also point out that activation of the classical and lectin pathways is impaired by the NS1 protein of WNV through direct interaction with C4. The binding of NS1 to C4 decreased C4b deposition and C3 convertase (C4b2a) activity [222]. In the absence of an alternative pathway for the complement system activation, WNV spreads to the CNS at an earlier time point and is associated with a reduced CD8+ T cell response. Interestingly, however, despite the lack of knowledge of the mechanism, activation of the alternative pathway during the initial phases of infection may induce inflammatory mediators that facilitate the virus crossing the blood–brain barrier. It is suggested that TNF-α production in peripheral lymphoid tissues modulates blood-brain barrier permeability and WNV neuroinvasion. It has also been observed that the interaction of WNV with C1q facilitates infection of the spleen by the virus, but interestingly this infection does not dramatically alter the induction of primary antiviral B and T lymphocyte immune responses to WNV [220].

## 3. Concluding Remarks and Future Perspectives

Viral diseases are a huge public health problem in every latitude and age group. The complement system is a part of the immune system that serves as a functional bridge between innate and adaptive immune responses. It plays a key role in the host defense against pathogens, and viral infections cause rapid activation of its cascades. In addition to this role, the complement system also has a significant role in virus neutralization and mitigation of infection. However, depending on the type of virus, the complement system can be overactivated, as seen in the four groups of viral diseases presented above. Overactivation of the complement system can lead to a maladaptive immune response and cytokine storm, exacerbation of the disease, and later dysfunction of many cells and organs leading to multiple organ failure and even death. Therefore, researchers are increasingly turning their attention to the inhibition of the complement system activity as a therapeutic agent to reduce the inflammatory response. The use of endogenous complement system inhibitors, the use of antibodies or antagonists that block key proteins of the complement system cascade or neutralize anaphylatoxins and their interaction with receptors (C3aR and C5aR) can protect the body from complement-dependent cytotoxicity. However, a complete blockade can impair its ability to remove pathogens and increase the risk of subsequent infections. Therefore, targeting the complement system, especially in the course of severe diseases with inflammatory conditions, should aim at balancing or controlling its activation with suppression of adverse effects but without nullifying its protective functions.

## Figures and Tables

**Table 1:** Factors regulating the activity of the complement.

Factor		Function	References
C1 Esterase Inhibitor (C1-INH)		Plasma serine proteinase inhibitor (serpin). Binds to activated C1r and C1s, irreversibly inhibiting their activity, inhibition of classical pathway. Inhibits MASP-1 and MASP-2	[26,27]
Factor I (FI)		Protease inactivating C4b and C3b with cofactors	[28]
Soluble Regulatory Proteins: C4b-Binding Protein and factor H (FH)		Cofactors for factor I. Accelerates the decomposition of the C4b2a and C3bBb complex. It is necessary for the regulation of C3 activity	[27,29,30,31]
Membrane Regulatory Proteins		Protect cells from complement mediated lysis	[28,32]
	Decay-accelerating Factor (DAF) (CD55)	Factor accelerating the decomposition of C3 and C5 convertases	
	Membrane cofactor protein (MCP, CD46)	Binds components C3b and C4b in the free state or in convertase.	
Properdin		Stabilizes C3 and C5 convertases	[33,34]
Soluble MAC Inhibitors			[27,32]
	Vitronectin	Binds MAC and prevents the complex from being inserted into the cell membrane	
	Clusterin	Inactivates MAC with vitronectin	
Membrane MAC Inhibitor CD59		The primary membrane-bound inhibitor of the MAC. It binds to C8 and C9, preventing the incorporation and polymerization of C9	[27,32]

**Table 2:** Complement evasion strategies used by selected viruses.

Disease	Virus	Family	Strategies Evasion	References
Influenza	Influenza viruses	*Orthomyxoviridae*	Virus acquires CD59 on the surface and inhibits C1q-mediated recognition of virions Inhibition of neutralization by blocking the interaction of C1q with antibodies bound to the viral surface Inability of human C3b to recognize the surface of the virus and its opsonization	[60,61,62]
Severe Acute Respiratory Syndrome (SARS) Middle East Respiratory Syndrome (MERS) Coronavirus disease (COVID-19)	SARS-CoV MERS-CoV SARS-CoV-2	*Coronaviridae*	No data	-
Viral Lower Respiratory Tract Illiness	Respiratory syncytial virus (RSV)	*Pneumoviridae*	Transcriptional regulation of complement proteins	[63]
Hepatitis B (HB)	Hepatitis B virus (HBV)	*Hepadnaviridae*	The HBV X protein (HBx) upregulates CD59 and C4b-binding protein α (C4BPα), which inhibit the formation of MAC and provides protection from complement-mediated cytolysis	[64,65]
Ebola Virus Disease (EVD)	Ebola virus (EBOV)	*Filoviridae*	No data	-
		*Flaviviridae*	Non-structural protein NS1 function as a regulator of the complement system. NS1 directly binds C4b binding protein (C4BP) on the surface of infected cells resulting in inhibition of complement activation in all pathways and MAC formation	[66]
Dengue	Dengue virus (DENV 1-4)		NS1 competitively binds to MBL, which prevents the later from recognizing and neutralizing the virus. NS1 binds clusterin/vitronectin on the surface of infected cells, resulting in the inhibition of complement activation in all pathways and MAC formation	[67,68,69]
Zika Virus Disease (ZVD)	Zika virus (ZIKV)		Incorporation into the viral envelope the of regulatory protein CD55 which contributes to virus stability and helps to avoid complement-dependent virolysis	[70]
West Nile Fever (WNF)	West Nile virus (WNV)		NS1 directly binds and recruits FH to the surface of infected cells resulting in the inhibition of complement activation in all pathways and MAC formation	[71]

## Data Availability

Not applicable.
